# Examining which clinicians provide admission hospital care in a high mortality setting and their adherence to guidelines: an observational study in 13 hospitals

**DOI:** 10.1136/archdischild-2019-317256

**Published:** 2020-03-12

**Authors:** Morris Ogero, Samuel Akech, Lucas Malla, Ambrose Agweyu, Grace Irimu, Mike English, Clinical Information Network Author Group, Victor Juma

**Affiliations:** 1 Health Services Unit, KEMRI-Wellcome Trust Research Programme, Nairobi, Kenya; 2 School of Mathematics, University of Nairobi College of Biological and Physical Sciences, Nairobi, Kenya; 3 Pediatrics, University of Nairobi, Nairobi, Kenya; 4 Nuffield Department of Medicine, University of Oxford, Oxford, UK

**Keywords:** general paediatrics, quality of care, care cascade, adherence to clinical guidelines

## Abstract

**Background:**

We explored who actually provides most admission care in hospitals offering supervised experiential training to graduating clinicians in a high mortality setting where practices deviate from guideline recommendations.

**Methods:**

We used a large observational data set from 13 Kenyan county hospitals from November 2015 through November 2018 where patients were linked to admitting clinicians. We explored guideline adherence after creating a cumulative correctness of Paediatric Admission Quality of Care (cPAQC) score on a 5-point scale (0–4) in which points represent correct, sequential progress in providing care perfectly adherent to guidelines comprising admission assessment, diagnosis and treatment. At the point where guideline adherence declined the most we dichotomised the cPAQC score and used multilevel logistic regression models to explore whether clinician and patient-level factors influence adherence.

**Results:**

There were 1489 clinicians who could be linked to 53 003 patients over a period of 3 years. Patients were rarely admitted by fully qualified clinicians and predominantly by preregistration medical officer interns (MOI, 46%) and diploma level clinical officer interns (COI, 41%) with a median of 28 MOI (range 11–68) and 52 COI (range 5–160) offering care per study hospital. The cPAQC scores suggest that perfect guideline adherence is found in ≤12% of children with malaria, pneumonia or diarrhoea with dehydration. MOIs were more adherent to guidelines than COI (adjusted OR 1.19 (95% CI 1.07 to 1.34)) but multimorbidity was significantly associated with lower guideline adherence.

**Conclusion:**

Over 85% of admissions to hospitals in high mortality settings that offer experiential training in Kenya are conducted by preregistration clinicians. Clinical assessment is good but classifying severity of illness in accordance with guideline recommendations is a challenge. Adherence by MOI with 6 years’ training is better than COI with 3 years’ training, performance does not seem to improve during their 3 months of paediatric rotations.

**What is already known on this topic?**

- Although most deaths are within 48 hours of admission in many low/middle-income country (LMIC) hospitals the proportion of cases preregistration clinicians admit has rarely been quantified.
- Paediatric hospital care in many LMICs can be of poor quality with limited adherence to guidelines and this may contribute to poor outcomes.
- Efforts to assess clinicians’ adherence to guidelines often evaluate care as isolated components rather than as correctly conducted, sequential steps for each individual.

**What this study adds?**

- Preregistration clinicians (interns) are responsible for the large majority of admissions to hospitals in high mortality settings that offer experiential training in Kenya.
- Perfect, sequential adherence to guidelines occurs in ≤12% children admitted with common illnesses, the major challenge is classification of illness severity.
- Although perfect guideline adherence is uncommon, most children are prescribed approved first-line treatment but this often represents overuse of treatment for severe illness.

## Background

In many African countries physician trainees (medical officer interns (MOI) in Kenya) and non-physician clinician trainees (clinical officer interns (COI) in Kenya) undertake a preregistration internship after preservice training of 6 years at a university and 3 years of technical college, respectively. Typically, they have between 10 and 18 weeks of preservice paediatric ward-based training. In Kenya, the 1-year internship is intended to be a period of hospital-based employment in 1 of over 70 eligible hospitals where practice is carefully supervised prior to professional registration. To provide supervision hospitals that serve as internship centres usually have registered general medical officer (MO) and clinical officer (CO) who have successfully completed internships) and should have at least one paediatrician overseeing all paediatric and neonatal hospital care. Anecdotally, however, initial care for children admitted to hospitals in many low and middle-income countries often seems to be provided by preregistration clinicians with senior clinicians, when available, often only reviewing patients much later. We aimed to explore how often admission care was provided by preregistration clinicians.

To promote a basic level of good practice in such hospitals, Kenya adapted WHO guidance for inpatient management of children^1 2^ to produce clinical protocols for common disorders that take account of the limited diagnostic and treatment resources available.^3^ Assessing adherence to these protocols is one means to assess the technical quality of initial clinical care.^4 5^ To facilitate this we recently developed the Paediatric Admission Quality of Care (PAQC) score.^6 7^ The PAQC score is a composite measure encompassing defined steps in the clinical history, physical examination, diagnosis and treatment that should be performed at admission and spans three common conditions—diarrhoea with dehydration, malaria and pneumonia. The score is intended to assess the quality of care at admission because most inpatient deaths occur within the first 48 hours.^8^

Since 2013, our team has also been working with multiple Kenyan hospitals that form the Clinical Information Network (CIN).^8–10^ The CIN has successfully fostered the implementation of standardised paediatric admission records (PAR) and creation of high-quality routine data on hospitals’ paediatric admissions.^9 11^ These data also allow us to assess how guideline-adherent care is, if there are specific challenges in guideline adherence and factors associated with reduced adherence. In this study we therefore aimed to address the following questions:

1. Which clinicians and how many are responsible for admission care in hospitals offering internship training in Kenya, and how many patients do they admit?
2. What proportion of patients are managed exactly in accordance with the key initial steps outlined in Kenyan clinical protocols?
3. Among children admitted by internship clinicians do factors such as clinician cadre influence adherence to guideline recommendations?

## Methods

### Study design and data collection

The Kenya Medical Research Institute/Wellcome Trust Research Programme in collaboration with the Kenya Paediatric Association, the University of Nairobi and the Kenya Ministry of Health (MoH) purposively recruited 13 county hospitals into the CIN.^12^ The basis of selection was to include rural and urban regions and varied geographical zones and malaria endemicity. These hospitals were selected from the group of 70 internship training hospitals. Interns in these hospitals typically rotate into the paediatric department for 3 months of the 12 months’ internship.

#### Admissions database

Children are admitted to the hospitals in CIN by the duty clinician who completes a structured paediatric admission record (PAR)^13^ capturing patient demographics, presenting symptoms and clinical signs and admission diagnoses; additional charts help identify diagnostic tests ordered and prescribed treatment. Upon discharge or death, a trained data clerk retrospectively abstracts these data, discharge diagnoses and outcome into a customised electronic data capture tool (Research Electronic Data Capture).^14^ A full description of the data collection procedures including training, periodic refresher courses for clerks, daily local monitoring of data quality, centralised data quality monitoring and the process of web-based data synchronisation is provided elsewhere.^10^

#### Clinician database

Since November 2015, the data clerks have also recorded basic details on all clinicians responsible for paediatric admissions. Data are recorded in a secure separate log and accompanying database. Each clinician is assigned a unique identifier linked to their gender, cadre (MO, CO), whether they were interns (MOI, COI) and their rotation start date (for MOI and COI). At the point of reviewing a patient’s file on discharge, the clerk identifies the admitting clinician and links the patient record to the clinician’s unique identifier.

### Study population

**Figure 1:**
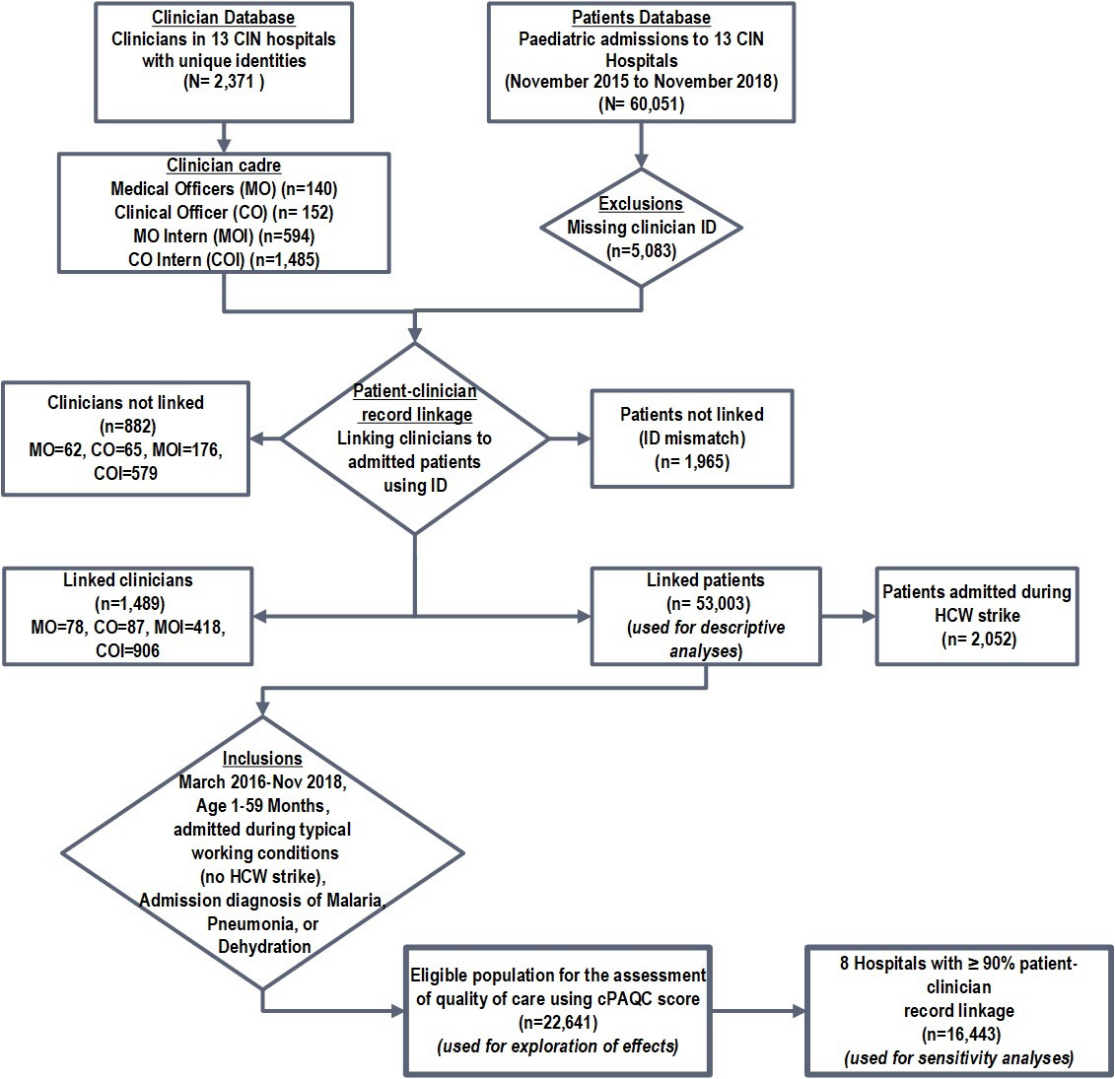
Study population. Success with patient–clinician record linkage varied across hospitals. For instance, in eight hospitals, over 90% of all patients were linked while in the remaining five hospitals linked patients ranged from 52.5% to 86.6%. Malaria was more common among patients not linked reflecting lower success at record linkage in hospitals in settings of high malaria endemicity. CIN, Clinical Information Network; cPAQC, correctness of Paediatric Admission Quality of Care; HCW, healthcare worker.

Patients’ records from paediatric wards of the 13 hospitals from November 2015 through November 2018 linked to an admitting clinician were used for descriptive analyses. Patients aged 1–59 months admitted from March 2016 (when national pneumonia guidelines changed) through November 2018 were eligible for inferential analyses if they had a clinical diagnosis of malaria, pneumonia or dehydration (figure 1). The period from December 2016 to November 2017 was marked by a series of prolonged nationwide health worker strikes^15^ and we refer to this period as a ‘strike year’.

All clinicians (represented by their unique identifier) admitting children during the study period were also potentially eligible for analyses of quality of care. We included clinicians identified as MOI or COI in analyses exploring whether cadre was associated with quality of care assessed using a modified PAQC score (see the section below). We further explored whether quality of care was associated with the ‘early’ (first 5 weeks) or ‘late’ (after 5 weeks) period of their ward rotation to see if practices changed during the 3-month internship rotation.

### Creating the cumulative correctness of Paediatric Admission Quality of Care score

The original PAQC score was designed to assess adherence to MoH Basic Paediatric Protocols for the three most common childhood admission diagnoses—malaria, pneumonia and diarrhoea with dehydration.^3 7^ It was based on three discrete domains (assessment, diagnosis/severity classification and treatment) that were allocated scores independently prior to summation. This scoring approach was amended for use in the current study for four reasons. First, the original score predated revised Kenyan and WHO pneumonia guidelines.^16^ Second, the improved routine data available from CIN meant that in the assessment domain the original 3-point score (0/1/2) could be simplified to a 2-point scale (0/1 representing complete/incomplete documentation of assessment) to minimise redundancy. Third, the data captured on the PAR also allowed us to derive from the data a ‘correct syndromic diagnosis’ according to the Kenyan protocols. This meant we could compare the clinician’s classification/diagnosis with what the protocol indicated was correct (and score classification correctness 0/1).

Lastly, we revised the scoring approach so that it reflected whether the clinicians’ practice was perfectly adherent across the sequence of correct assessment, classification, treatment choice (for disease and classification) and treatment dose after excluding patient groups with different treatment needs (as shown in online supplementary file 4). This means one can only get a correct score in a later step of this sequence if all of the previous steps are also correct. These revisions (outlined in online supplementary file 1) resulted in the creation of the correctness of Paediatric Admission Quality of Care (cPAQC) score on a 5-point scale (0–4) with a maximum score representing perfect correctness and other scores representing the point in the sequence of steps where a clinician deviated from perfect adherence. For patients with multimorbidities, we used an all-or-none combination of disease-specific step scores as in the original PAQC score.^7^ This means if a patient has malaria and pneumonia they must perfectly adhere to both protocols, each in sequence, to get a maximum score.

10.1136/archdischild-2019-317256.supp1 Supplementary data

10.1136/archdischild-2019-317256.supp2 Supplementary data

For subsequent exploratory modelling we reviewed the cPAQC scores and identified that the largest drop in performance occurred at the severity classification stage and so considered a cPAQC score ≥2 to represent guideline-adherent care and a score of ≤1 to represent non-adherent care.

### Statistical analyses

We use descriptive statistics for patient and clinician characteristics reporting these as frequencies (%) and median and IQRs as appropriate. To explore whether clinician-level factors including cadre and whether the early or late phase of a clinician’s internship affect the quality of care (guideline-adherent vs non-adherent care) we used Bayesian hierarchical models using *Stan* 17 and the Hamiltonian Monte Carlo approach which has advantages in fitting complex models^18^ (see online supplementary file 2 for details including findings of a sensitivity analysis). Significance of ORs was assumed if 95% credible intervals excluded one. All analyses were performed using R V.3.4.3 (R Foundation for Statistical Computing, Vienna, Austria; http://www.cran.r-project.org).

10.1136/archdischild-2019-317256.supp3 Supplementary data

## Results

### Data linkage

The full study cohort included 60 051 patients admitted to paediatric wards of the participating hospitals and a total of 2371 clinicians (figure 1). Linking the two databases (patient and clinician) was not possible for a total of 7048 patients and 881 clinicians (figure 1). Consequently, only 53 003 patients linked to 1489 clinicians of various cadres were eligible for further analyses; 2052 (3.88%) patients were admitted in the strike year (figure 1).

### Characteristics of study population

Out of the eligible population (n=53 003), there were 23 547 (44.59%) females, median age was 17 (IQR 7–42) months and 3199 (6.03%) patients died. Pneumonia was the single most common admission diagnosis. Patient characteristics were not appreciably different between linked and unlinked patients (table 1).

**Table 1:** Distribution of patient characteristics across groups

Patient characteristics	Linked (n=53 003)	Not linked (n=7048)
Mortality	3199 (6.03%)	449 (6.42%)
Age (months), median (IQR)	17 (7–42)	24 (10–57)
Gender (female)	23 547 (44.59%)	3078 (44.17%)
Weight (kg), median (IQR)	9 (6–14)	10 (8–16)
Fever	31 096 (72.48%)	4025 (75.15%)
Unresponsive on AVPU scale	457 (1.08%)	57 (1.08%)
Malaria	10 831 (20.43%)	2285 (32.42%)
Pneumonia	20 267 (38.24%)	2084 (29.57%)
Dehydration	9412 (17.76%)	1181 (16.76%)
One comorbidity	29 738 (81.67%)	3874 (77.31%)
Two comorbidities	6359 (17.46%)	1073 (22.41%)
Three comorbidities	314 (0.86%)	64 (1.28%)

AVPU, alert verbal pain unresponsive.

### Workload of the admitting clinicians

**Figure 2:**
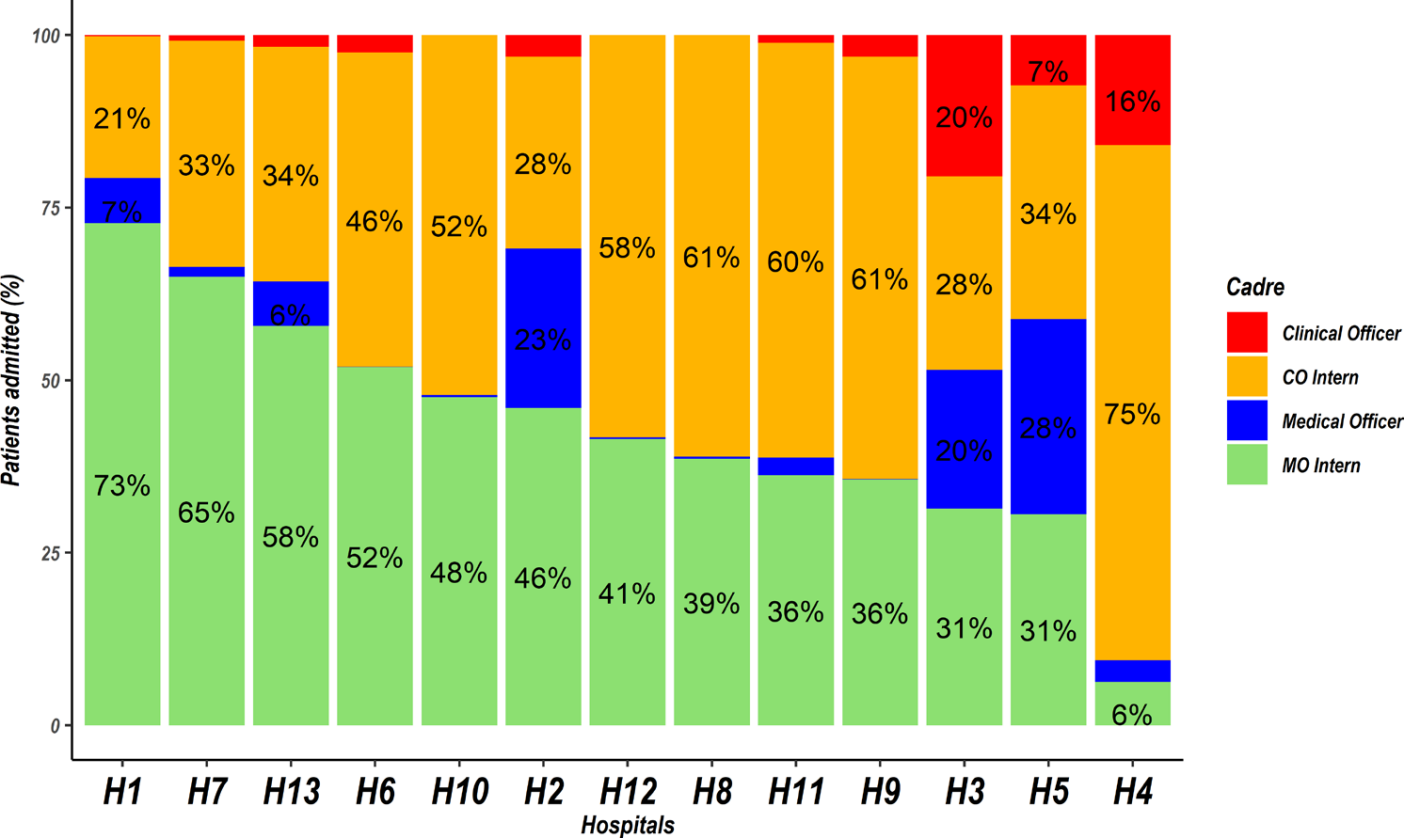
Patients admitted by clinicians of various cadres across hospitals. Hospitals are arranged from left to right in the descending order according to the proportion of patients admitted by medical officer interns (MOI). Red and blue bars without values represent cadres whose admissions were <4% in a given hospital. CO, clinical officer; MO, medical officer.

In the 25-month non-strike period there were 1489 clinicians who were successfully linked to the eligible patient population, 869 (58.30%) clinicians were male and COI and MOI comprised the largest groups, with median (range) numbers per hospital of 52 (5–160) COIs and 28 (11–68) MOIs over this period. Far fewer fully registered practitioners were involved in admitting children (table 2) although patterns for which cadre was responsible for admitting most children varied quite substantially across hospitals (figure 2). The MOI group admitted the greatest number of children closely followed by the COI group (46.1% and 40.9% of all linked cases respectively (see table 2 and figure 2)). There was a considerable decrease in the number of patients clinicians admitted during the strike year (table 2).

**Table 2:** Comparison of patient admission workload for different cadres and between the strike year and a period without healthcare workers’ strikes

Patients admitted in the non-strike period (n=44 719)				Patients admitted during strike year (December 2016 to November 2017) (n=8284)		
Clinician cadre	Median number of clinicians per cadre (IQR)	Median number of patients per cadre (IQR)	Median number of patients per each clinician (IQR)	Median number of clinicians per cadre (IQR)	Median number of patients per cadre (IQR)	Median number of patients per each clinician (IQR)
Clinical officer	6 (2–14)	47 (11–157)	10 (3–24)	3 (1–4)	3 (0–24)	4 (1–10)
CO intern	46 (41–86)	1328 (995–1579)	13 (4–29)	15 (9–30)	213 (127–416)	5 (2–17)
Medical officer	5 (2–8)	82 (5–501)	17 (2–73)	1 (1–5)	5 (1–20)	4 (1–11)
MO intern	27 (22–34)	1436 (999–1660)	40 (9–78)	10(7–13)	290 (164–466)	10 (2–48)

CO, clinical officer; IQR, interquatile range; MO, medical officer.

### Quality of care at admission

**Figure 3:**
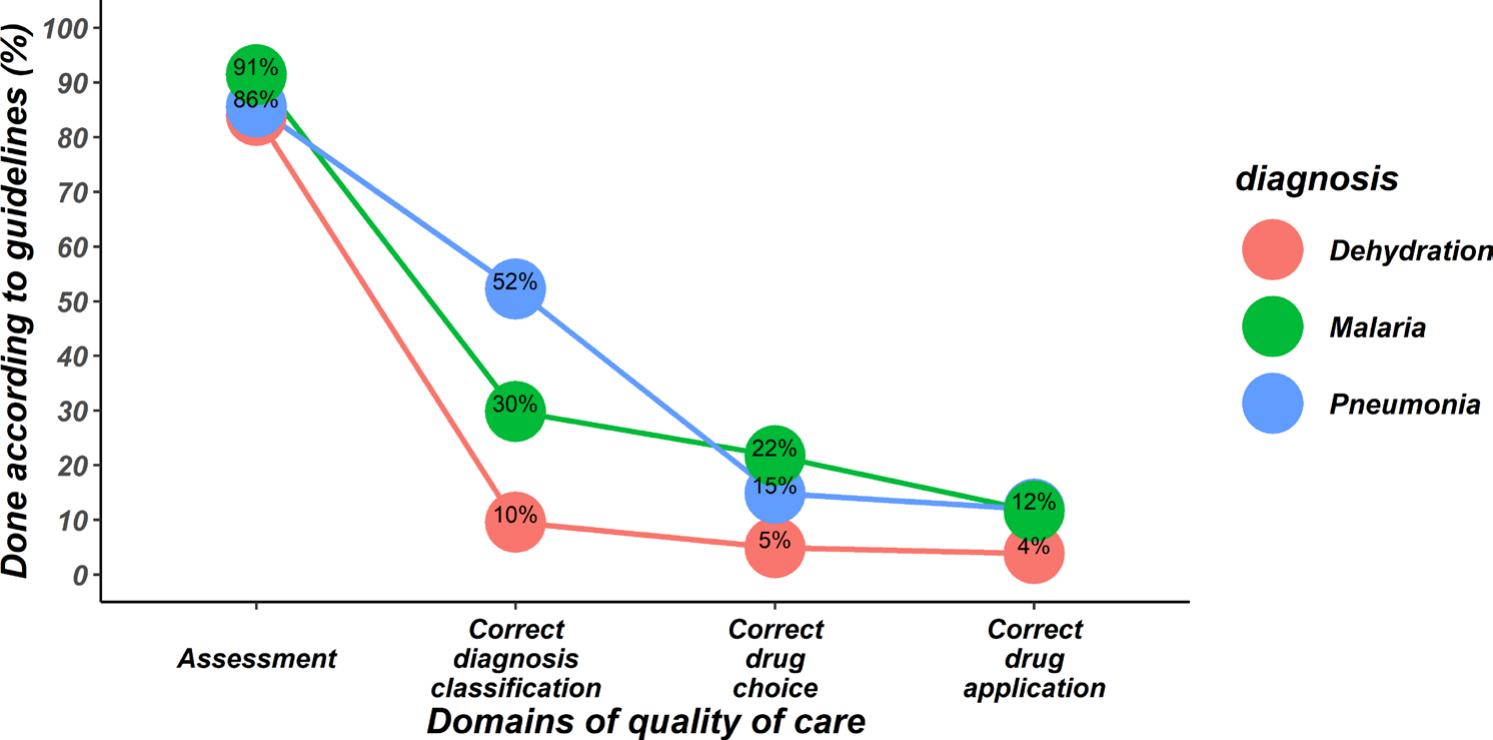
Performance of items constituting the correctness of Paediatric Admission Quality of Care (cPAQC) score for patients with diarrhoea/dehydration, malaria and pneumonia as assessed for medical officer interns (MOI). The cPAQC score spans four items of a care cascade such that correct performance of steps later in the pathway is only possible if earlier steps are also correct (represented as progression from left to right on the X-axis where axis labels also represent progression of the cPAQC score from 1 to 4). Performance is represented as the percentage of the 10 115 patients (admitted by MOI) who achieved cPAQC scores for the respective diagnoses of 1, 2, 3 or 4.

To explore quality of care, we analysed data from 22 641 eligible patients using the cPAQC score (figure 1). We present illustrative results for MOI who admitted the most patients (n=10 115). We note a substantial drop in the cPAQC score at the point of severity classification for each disease. This indicates that while clinicians typically document a full patient assessment their classification is then often not correct as judged by the Kenyan protocols (see figure 3). For pneumonia where correct classification is more common there is a further marked decline in cPAQC score because the treatment prescribed is not correctly aligned with the disease classification that the recorded clinical signs and agreed protocol recommend. The end result is that perfect, sequential adherence to protocols is found in 12% or less of children admitted with malaria, pneumonia or diarrhoea with dehydration. Similar cascades for 9891 children admitted by COI, 1617 children admitted by MO and 1018 children admitted by CO were similar to those presented for MOI (online supplementary file 3).

10.1136/archdischild-2019-317256.supp4 Supplementary data

While perfect adherence to Kenyan protocols was relatively poor additional analyses indicated that among children with pneumonia 74.4% (6636/8921) were prescribed one of the two recommended first-line treatments (amoxicillin or penicillin with gentamicin) overall and when these drugs were prescribed in 84.5% (5605/6636) cases doses were correct. Similarly, 82.9% (4882/5888) children with malaria and 66.1% (1912/2893) with diarrhoea with dehydration received a recommended first-line treatment for their disease (artemether-lumefantrine/artesunate or oral/intravenous fluids, respectively) and these prescriptions were of the correct dose in 57.9% (2826/4882) and 66.3% (1267/1912) prescriptions, respectively.

### Factors influencing guideline-adherent care at admission

We used data on 19 072 patients admitted by MOI or COI to explore factors associated with guideline-adherent care (table 3, with similar findings in our sensitivity analysis in online supplementary file 2). Patients with multimorbidity had significantly lower guideline adherence; two comorbidities (adjusted OR (AOR) 0.12 (95% CI 0.10 to 0.14)), three comorbidities (AOR 0.02 (95% CI 0.01 to 0.05)). Clinical data indicating the presence of any severe disease classification were associated with better guideline adherence (AOR 1.82 (95% CI 1.68 to 1.96)). MOI provided more guideline-adherent care compared with COI (AOR 1.19 (95% CI 1.07 to 1.34)) and care provided by clinicians in the later part of their rotation was marginally more likely to be guideline adherent (AOR 1.09 (95% CI 1.02 to 1.18)). Neither the child’s nor the clinician’s gender had an effect on guideline adherence, but younger patients aged 1–11 months were more likely to receive guideline-adherent care (AOR 1.21 (95% CI 1.13 to 1.30)).

**Table 3:** Results of multivariable model showing the degree to which clinician and patient factors (represented by adjusted odds ratios and 95% CI) are associated with a cPAQC score of ≥2 used as an indicator for more guideline-adherent care (n=19 072)

Covariate	AOR	95% credible intervals
Comorbidities		
One	Ref	
Two	0.12	0.10 to 0.14*
Three	0.02	0.01 to 0.05*
Clinician cadre		
COI	Ref	
MOI	1.19	1.07 to 1.34*
Practice period		
Early	Ref	
Late	1.09	1.02 to 1.18*
Illness severity classification		
Non-severe	Ref	
Severe	1.82	1.68 to 1.96*
Clinician gender		
Male	Ref	
Female	1.02	0.92 to 1.13
Child sex		
Female	Ref	
Male	1.01	0.95 to 1.09
Child age (months)		
1–11	Ref	
12–59	1.21	1.13 to 1.30*

*Denotes a statistically significant relationship where <1 means less guideline adherent and >1 means more guideline adherent.

AOR, adjusted odds ratio; COI, clinical officer intern; cPAQC, correctness of Paediatric Admission Quality of Care; MOI, medical officer intern.

## Discussion

In data from 13 county hospitals that train physician and non-physician clinician interns in Kenya, 1489 clinicians could be linked to 53 003 admissions over a period of 3 years including a period spanning major strikes. These interns rather than fully licensed clinicians admit 85% children. Each MOI admitted approximately twice as many patients as a COI during typical 3 months’ rotations. Reasons for this difference may be because COIs are typically unpaid and therefore may not work at nights or weekends, or because MOIs may have higher status and so they are preferentially given or take this work linked to the expectation that COs’ ultimate work is predominantly in ambulatory care.

Our data indicate that in the CIN hospitals there is a culture of good documentation of clinical symptoms and signs by interns (figure 3 and online supplementary material file 3), we believe inculcated and reinforced by senior clinical staff.^19 20^ We explored using a strictly defined, sequential score (the cPAQC score) at which steps in the process of care guideline adherence performance declines. We observed that clinicians commonly do not classify the severity of illness in accordance with guidance. Most often clinicians appear to overdiagnose severe illness. Potential explanations are that clinicians may follow a gut feeling about illness severity,^21^ although those studied had limited prior experience, or that they are risk averse and inclined to justify prescription of more aggressive treatment, an idea supported by prior work examining treatment allocation for pneumonia.^22^ A heightened concern for risk might perhaps explain the association of poor adherence with multimorbidity.^23 24^ Overtreatment as a result of overdiagnosing may be both wasteful and potentially could cause harm, for example, overuse of intravenous fluids in settings with poor inpatient monitoring.^25^

Among those admitted by internship clinicians our data suggest no effect of clinician gender on adherence to guidelines in keeping with other studies.^26 27^ There may be a small effect of cadre, with MOI more adherent than COI, and a marginal effect of learning during the interns’ 3 months’ rotation.^26 28^ The relatively weak effect of time period on guideline adherence may be because this is too short a period in which to observe individual learning, although it is notable that these interns adopted good documentation practices early in their internship. More probably it may be that those providing immediate supervision (typically MOs) may also not fully adhere to guidelines (as our data suggest) while supervision from paediatricians is limited.

### Limitations

This study had a number of limitations. All hospitals were public hospitals but there were still variations in patterns of staffing and workloads. We focus only on immediate admission care and rely on the documentation in the medical files to assess adherence and thus assume that lack of documentation equates to non-adherent care. We are therefore unable to capture any nuances in the clinical condition that might better explain admission treatment decisions. We also assume that these admitting clinicians should strictly follow the national guidelines and that any deviation from this is incorrect. Perfect adherence to admission guidelines represents only one technical aspect of much wider issues in quality care that our analyses cannot address. Lastly, clinician–patient record linkage was not 100%. That said, we believe that studying a large population across multiple settings over a period of 3 years provides some useful insights into who provides routine patient care and where clinicians appear to deviate most from recommendations.

## Conclusion

We present what we believe is the first study to report how much admission care is provided by junior, preregistered clinicians during their 3 months’ internship in an important group of Kenyan hospitals. While documentation practices are now good (the first step in our care cascade) classification of the level of severity of illness frequently deviates from guideline recommendations. At present there is little evidence that non-adherence is corrected during clinicians’ internships raising questions about their supervision. More encouraging is the observation that the majority of children are at least prescribed correctly one of the recommended first-line treatment strategies for pneumonia, malaria and diarrhoea with dehydration. However, as the most common deviation from guidelines is overdiagnosis of severe illness it is possible that resources are being wasted and that children may spend more time in hospital receiving treatments that might cause harm or discomfort (eg, intravenous drugs). Further studies should explore the reasons for non-compliance, the possible consequences of overuse of treatments and whether de-escalating treatment is beneficial or harmful.
