# Supplementary material for: Examining which clinicians provide admission hospital care in a high mortality setting and their adherence to guidelines: an observational study in 13 hospitals
Source: Arch Dis Child. 2020 Mar 12;105(7):648–54. doi: 10.1136/archdischild-2019-317256 (PMC7361020; doi:10.1136/archdischild-2019-317256)

Figure 1: Logic used for Malaria patients

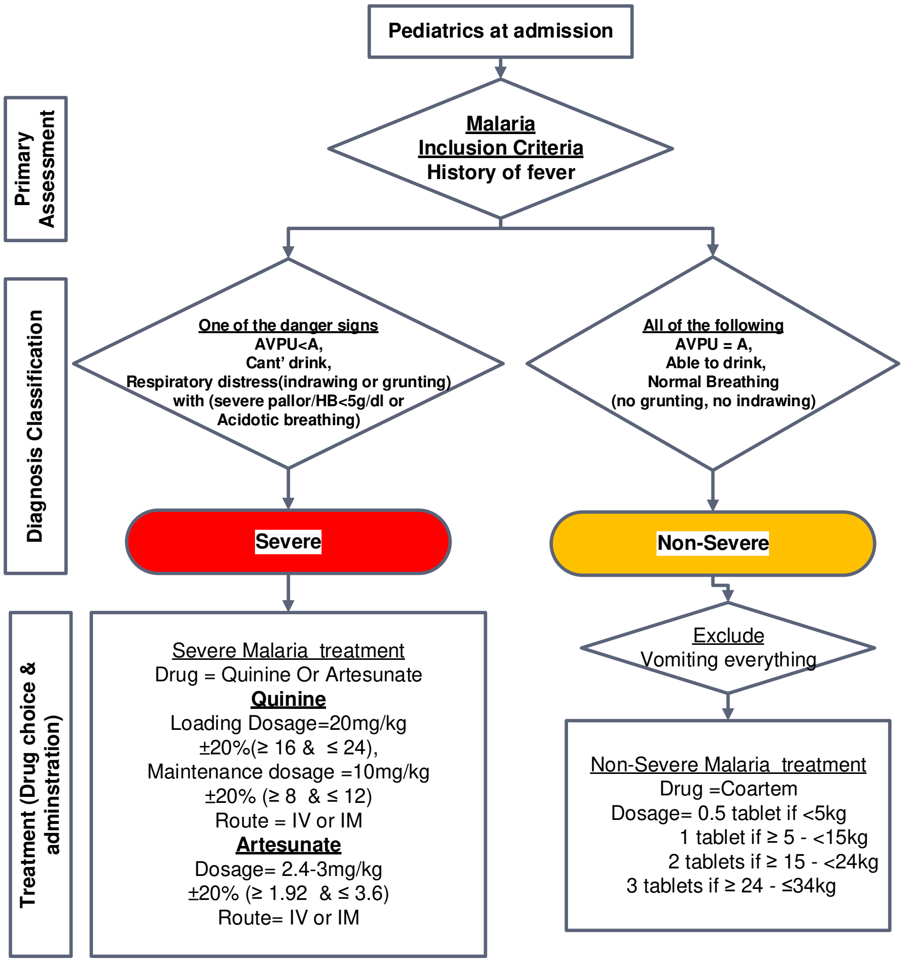

Figure 2: Logic used for Pneumonia patients

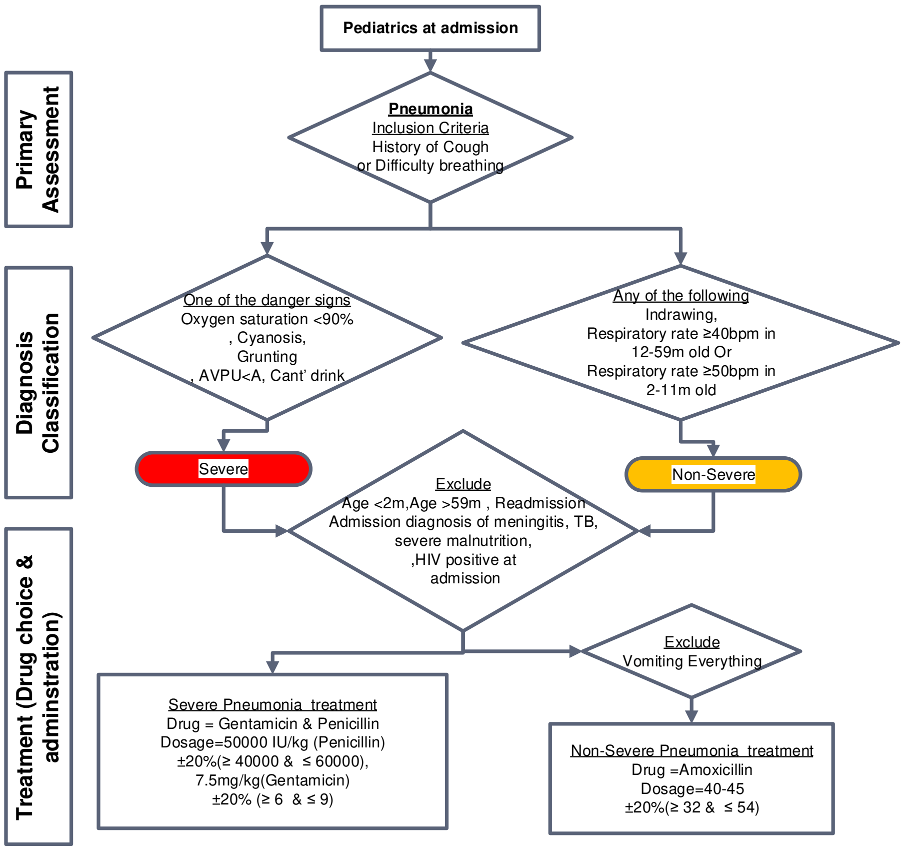

Figure 3: Logic used for diarrhea/dehydration patients

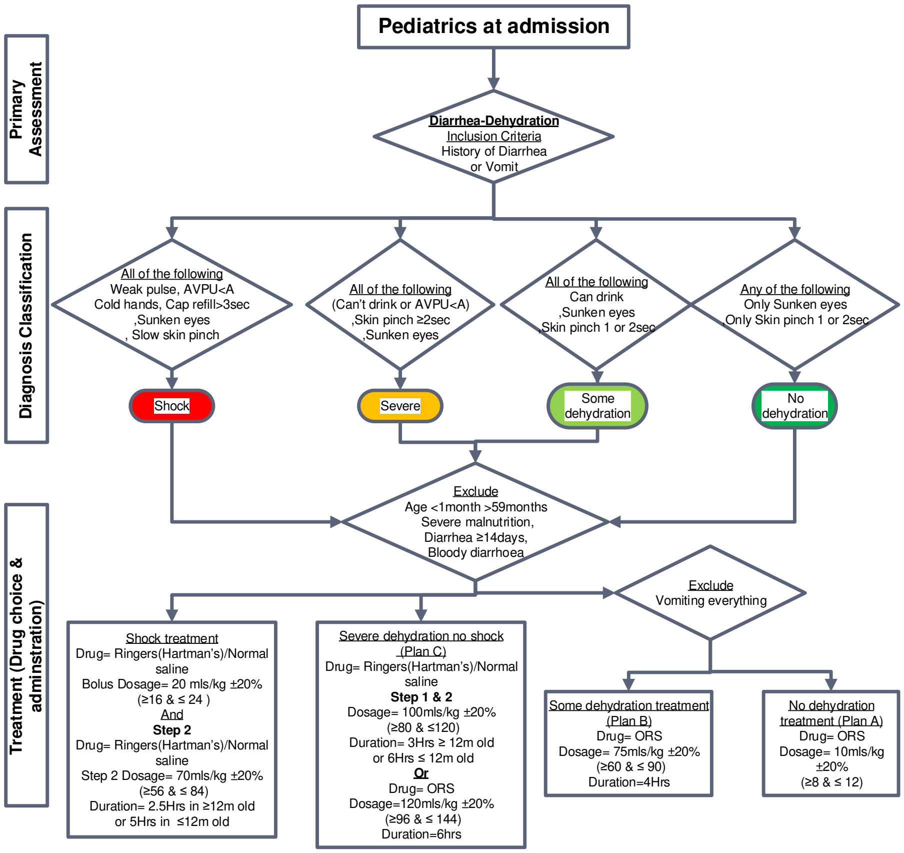

Supplement: Supplementary data [file archdischild-2019-317256supp001.pdf]
