# Supplementary material for: Examining which clinicians provide admission hospital care in a high mortality setting and their adherence to guidelines: an observational study in 13 hospitals
Source: Arch Dis Child. 2020 Mar 12;105(7):648–54. doi: 10.1136/archdischild-2019-317256 (PMC7361020; doi:10.1136/archdischild-2019-317256)
Supplement: Supplementary data [file archdischild-2019-317256supp002.pdf]

Appendix Table 1: Malaria Cumulative Correctness of Pediatric Admission Quality of Care (cPAQC) score allocation table

**Malaria**			
**Domain**	**Domain details**	**Clinical task**	**Scoring criteria**
Primary assessment	Fever documentation	Assess fever and document	Score 1 if all items in Primary & secondary domains are documented
Secondary assessment	Convulsions documentation	Assess all signs and document	
	Acidotic breathing documentation		
	Ability to drink documentation		
	AVPU documentation		
	Pallor documentation		
	Grunting documentation		
	Indrawing documentation		
Complete assessment	Primary & secondary assessment	Complete documentation of primary and secondary signs of malaria	
Diagnosis classification	**Severe malaria**		
	Fever plus any of the following:	Use clinical signs to classify malaria severity levels accordingly	Score 1 if there is a complete assessment of signs and malaria severity classification done according to guidelines
	AVPU<A		
	Inability to drink		
	Respiratory distress with severe anemia		
	Acidotic breathing		
	**Non- Severe malaria**		
	Fever plus all of the following:		
	AVPU=A		
	Can drink/breastfeed		
	No grunting		
	No indrawing		
Drug choice	**Severe malaria drugs**	Malaria drug prescriptions	Score 1 if preceding clinical tasks and drug(s) are prescribed according to guidelines.
	Artesunate or		
	Quinine		
	**Non-severe malaria drug**		
	Coartem		

Drug use/application	**Severe malaria treatment**	Apply malaria drugs appropriately (correct dosage and frequency)	Score 1 if preceding clinical tasks are done according to guidelines and drugs applied appropriately
	Dose: Quinine loading dose >=16 - 24mls/kg		
	Dose: Quinine maintenance dose >=8 - 12 mls/kg<br>Frequency: 8hourly		
	Dose: Artesunate >= 1.92 - 3.6mls/kg		
	**Non-severe malaria treatment**		
	Dose:<br>0.5 tablet of coartem for <5kgs<br>1 tablet of coartem for 5-15kgs<br>2 tablets of coartem for 15-24kgs<br>3 tablets of coartem for 24-34 kgs		
			**Maximum Malaria cPAQC Score=4**

Appendix Table 2: Pneumonia Cumulative Correctness of Pediatric Admission Quality of Care (cPAQC) score allocation table

Pneumonia			
**Domain**	**Domain details**	**Clinical task**	**Scoring criteria**
Primary assessment	Cough OR difficult breathing	Assessment and documentation of cough or difficult breathing	Score 1 if all items in Primary & secondary domains are documented
Secondary assessment	Cyanosis	Assess all signs and document	
	Ability to drink		
	AVPU		
	Respiratory rate		
	Grunting		
	Indrawing		
Complete assessment	Primary & secondary assessment	Complete documentation of primary and secondary signs of pneumonia	
Diagnosis classification	**Severe Pneumonia**	Use clinical signs to classify pneumonia severity levels accordingly	Score 1 if there is a complete assessment of signs and pneumonia severity classification done according to guidelines
	Cough or difficult breathing plus any of the following:		
	Oxygen saturation< 90%		
	Cyanosis		
	Grunting		
	Grunting		
	AVPU<A		
	Inability to drink		
	**Non- Severe Pneumonia**		
	Cough or difficult breathing plus any of the following:		
	Indrawing,		
	Respiratory rate >=40 in 12-59 months or Respiratory rate >=50 in 2-11 Months		
Drug choice	**Severe pneumonia drugs**	Pneumonia drug prescriptions	Score 1 if preceding clinical tasks and drug(s) are prescribed according to guidelines
	Penicillin and		
	Gentamicin		

	**Non-severe pneumonia drug**		
	Amoxil		
Drug use/application	**Severe pneumonia treatment**	Apply pneumonia drugs appropriately (correct dosage, frequency and route)	Score 1 if preceding clinical tasks are done according to guidelines and drugs applied appropriately
	Dose: Penicillin >= 40000-60000 IU/kg<br>Route: IV or IM		
	Dose: Gentamicin >= 6-9 MU/kg<br>Route: IV or IM		
	**Non-severe pneumonia treatment**		
	Dose: Amoxil >= 32-54mg/kg		
			**Maximum Pneumonia cPAQC Score=4**

Appendix Table 3: Dehydration Cumulative Correctness of Pediatric Admission Quality of Care(cPAQC) score allocation table

Dehydration			
**Domain**	**Domain details**	**Clinical task**	**Scoring criteria**
Primary assessment	*History of diarrhea OR Vomits*	Assessment and documentation of history of diarrhea or vomits	Score 1 if all items in Primary & secondary domains are documented
Secondary assessment	Capillary refill	Assess all signs and document	
	Temperature gradient		
	Sunken eyes		
	Skin pinch		
	Ability to drink		
	AVPU		
Complete assessment	Primary & secondary assessment	Complete documentation of primary and secondary signs of illness	
Diagnosis classification	**Severe dehydration**	Use clinical signs to classify dehydration severity levels accordingly	Score 1 if there is a complete assessment of signs and pneumonia severity classification done according to guidelines
	History of diarrhea OR Vomits plus all of the following:		
	Inability to drink OR AVPU<A		
	Skin pinch >=2 seconds		
	Sunken eyes		
	**Some dehydration no shock**		
	History of diarrhea OR Vomits plus all of the following:		
	Can drink/breastfeed		
	Sunken eyes		
	Skin pinch 1-2 seconds		
	**No dehydration**		
	History of diarrhea OR Vomits plus any of the following:		
	Sunken eyes AND Skin pinch is immediate		
	Skin pinch 1-2 seconds		
Drug choice	**Severe dehydration drug**		

	Ringers (Hartman's) or Normal saline or ORS (no IV line)	Dehydration drug prescriptions	Score 1 if preceding clinical tasks and drug(s) are prescribed according to guidelines
	**Some dehydration drug**		
	ORS		
	**No dehydration drug**		
	ORS		
Drug use/application	**Severe dehydration treatment**	Apply dehydration drugs appropriately (correct dosage, frequency and duration)	Score 1 if preceding clinical tasks are done according to guidelines and drugs applied appropriately
	Drug= Ringers(Hartman's)/Normal saline<br>Step 1 & 2<br>Dosage= 100mls/kg ±20% (≥80 & ≤120)<br>Duration= 3Hrs ≥ 12m old or 6Hrs ≤ 12m old<br>**OR**<br>Drug= ORS<br>Dosage=120mls/kg ±20% (≥96 & ≤ 144)<br>Duration=6hrs		
	**Some dehydration treatment**		
	Dosage ORS: >=60 - <= 90<br>Duration: 4Hrs		
	**No dehydration treatment**		
	Dosage ORS: >= 8 - <= 12		
			**Maximum dehydration cPAQC Score=4**
