# Supplementary material for: Examining which clinicians provide admission hospital care in a high mortality setting and their adherence to guidelines: an observational study in 13 hospitals
Source: Arch Dis Child. 2020 Mar 12;105(7):648–54. doi: 10.1136/archdischild-2019-317256 (PMC7361020; doi:10.1136/archdischild-2019-317256)
Supplement: Supplementary data [file archdischild-2019-317256supp003.pdf]

Model specification and selection

We initially used hierarchical logistic regression models with a 3-level structure: admitted patients (level 1); admitting clinicians (level 2); and hospitals (level 3). Variables included in the models were: patient's age; child sex; number of comorbidities (1, 2, 3); illness severity level (severe, non-severe); clinician cadre; clinician gender and internship practice period (early (first five weeks), late (last 7 weeks)). Since multicollinearity was not a concern and missing data in all these explanatory variables were ignorable (<1%), we proceeded to perform a complete case analysis. However, these models failed to converge due to complexity in estimation of likelihoods when using the R package, lme4.

To determine the optimal variance structure of the model, we explored a total of 4 a-priori likely models presented in Table 1. Each of these models had similar explanatory variables but with varying variance structure (random effect part).

Table 1: model selection

Model name	Model Description
• Model 1	Model with only fixed effects.
• Model 2	Model 1 + hospitals as random effects.
• Model 3	Model 1 + clinicians as random effects.
• Model 4	Model 1 + nested random effects (different intercepts for each clinician within hospital)

For all models, we specified 4 chains which is considered adequate, each with 2000 iterations, half of which were devoted to the warm-up (adjusting the behaviour of the sampler) and were automatically discarded before results were displayed. To promote good mixing Hamiltonian Monte Carlo (HMC) chains, as recommended, we used weakly informative priors. Moreover, these priors are not very sensitive, in that, reasonable changes in the prior do not produce noticeable changes in the posterior [1]. To assess model convergence, we performed Gelman-Rubin diagnostics[2] which includes visual inspection of

the model chains of the estimated parameters. On convergence, all four chains of the samples should intermingle well and look highly similar to one another [3].

We compared candidate models in Table 1 using the recommended approximated leave-one-out cross-validation [4, 5] and the result suggested that model 4 best fitted the data. The chosen model is an adjusted hierarchical logistic regression model which allowed for clustering of patients by clinicians nested within different hospitals. Convergence of this model shown in Figure 1 suggested that all 4 chains converged. Posterior predictive checks of the same model were done by graphically comparing the densities of the actual data and the data replicated from the models' posterior distribution and the result (see Figure 2) suggested that these densities were almost identical. Therefore, we proceeded to make inference.

Sensitivity analysis

In order to examine the consistency of our model results, we replicated the above analysis to a data subset in which ≥90% patients could be linked to a specific clinician ID within each hospital.

Sensitivity analysis. Estimates of the factors influencing guideline-adherence in care using data from hospitals with only >= 90% patient-clinician record linkage (n=13438)

Covariate		AOR	95% Credible intervals
Comorbidities	One	ref	
	Two	0.10	0.08-0.12*
	Three	0.02	0.01-0.08*
Clinician Cadre	COI	ref	
	MOI	1.19	1.05-1.35*
Practice Period	Early	ref	
	Late	1.12	1.01-1.24*

Illness Severity			
levels	Non-severe	ref	
	Severe	2.01	1.84-2.20*
Clinician Gender	Male	ref	
	Female	1.01	0.90-1.15
Child sex	Female	ref	
	Male	0.99	0.91-1.08
	12-59 months	ref	
Child age	1-11 months	1.24	1.14-1.35*

***denotes a statistically significant relationship where (<1 means less guideline adherent, > 1 more guideline adherent. AOR= Adjusted odds ratio**

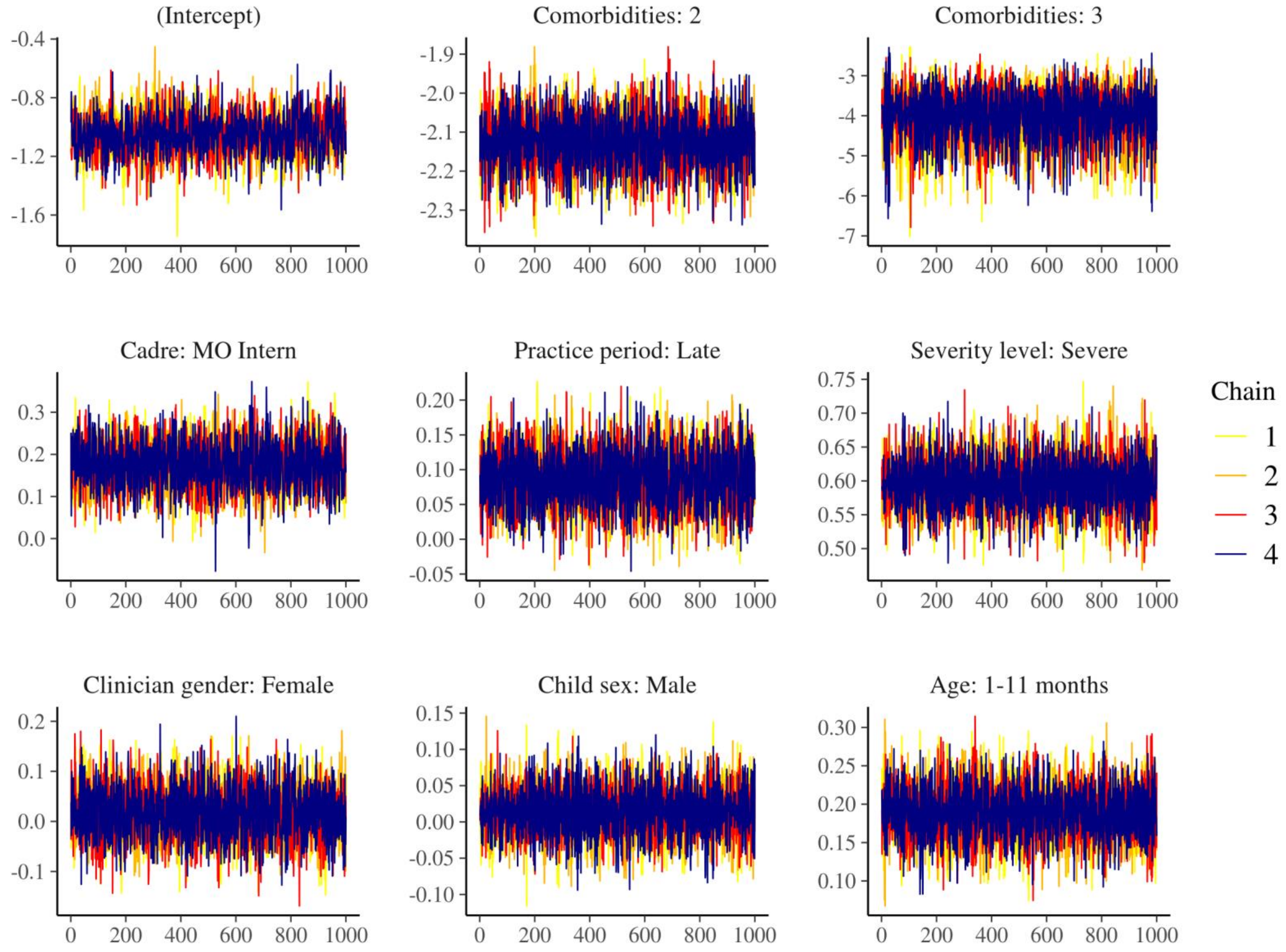

Figure 1: Trace plot of the chosen model. All four chains of the samples for each parameter look highly similar to one another an indicator of model convergence.

Ogero M, *et al. Arch Dis Child* 2020; 105:648–654. doi: 10.1136/archdischild-2019-317256

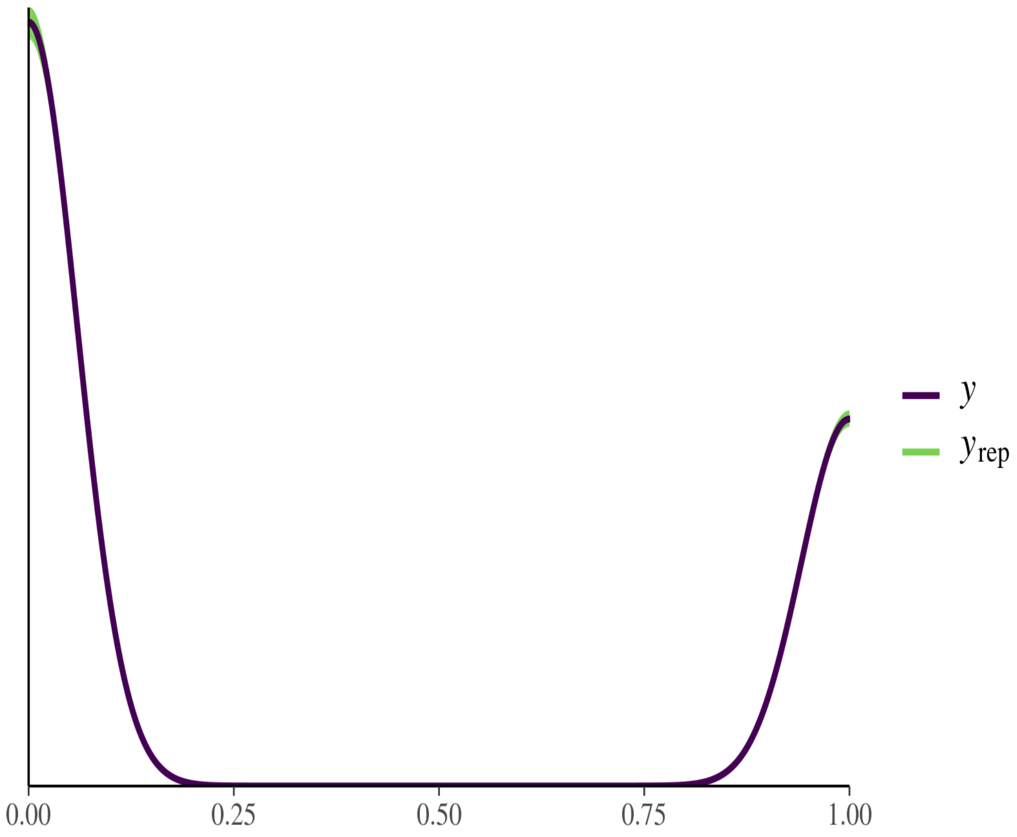

Figure 2: The plot of the posterior predictive density of the chosen model. *y* is the density of the observed data *yrep* is replicated data from the model. Densities of the observed and the replicated data look identical -an indicator of good fit.

Reference

1. Gelman, A., D. Lee, and J. Guo, *Stan: A probabilistic programming language for Bayesian inference and optimization.* Journal of Educational and Behavioral Statistics, 2015. **40**(5): p. 530-543.
2. Brooks, S.P. and A. Gelman, *General methods for monitoring convergence of iterative simulations.* Journal of computational and graphical statistics, 1998. **7**(4): p. 434-455.
3. Gelman, A., et al., *Bayesian data analysis*. 1995: Chapman and Hall/CRC.
4. Vehtari, A., A. Gelman, and J. Gabry, *Efficient implementation of leave-one-out cross-validation and WAIC for evaluating fitted Bayesian models.* arXiv preprint arXiv:1507.04544, 2015.
5. Vehtari, A., A. Gelman, and J. Gabry, *Practical Bayesian model evaluation using leave-one-out cross-validation and WAIC.* Statistics and Computing, 2017. **27**(5): p. 1413-1432.

Ogero M, *et al. Arch Dis Child* 2020; 105:648–654. doi: 10.1136/archdischild-2019-317256
