# Supplementary material for: Examining which clinicians provide admission hospital care in a high mortality setting and their adherence to guidelines: an observational study in 13 hospitals
Source: Arch Dis Child. 2020 Mar 12;105(7):648–54. doi: 10.1136/archdischild-2019-317256 (PMC7361020; doi:10.1136/archdischild-2019-317256)
Supplement: Supplementary data [file archdischild-2019-317256supp004.pdf]

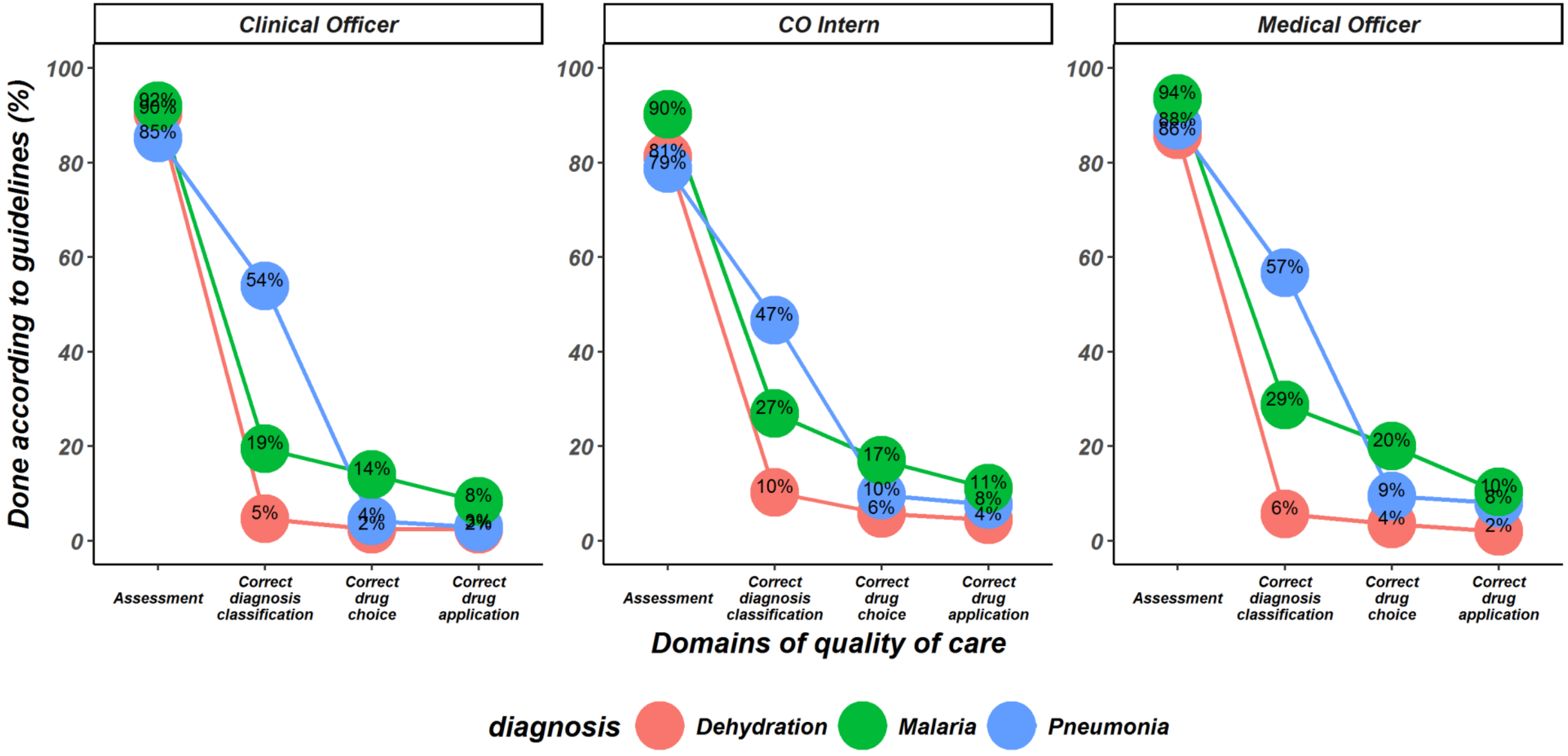

Figure 1: Performance of items constituting the cPAQC score for malaria, pneumonia and diarrhoea / dehydration patients as assessed for Medical Officer (MO) Clinical officer (CO), Clinical Officer intern(COI). The cPAQC score spans 4 items of a care cascade such that correct performance of steps later in the pathway is only possible if earlier steps are also correct (represented as progression from left to right on the X axis and equal to a cPAQC score of 1 to 4). Performance is represented as the percentage of the 22,641 patients who achieved scores for the respective diagnoses of 1, 2, 3 or 4.
